# Stroke-point-of-care ultrasound: a new holistic approach to bedside evaluation in stroke patients using ultrasound

**DOI:** 10.1093/esj/aakag027

**Published:** 2026-04-07

**Authors:** Claudio Baracchini, Alessio Pieroni, Filippo Maria Farina, Nicola Carraro, Piergiorgio Lochner, Caterina Kulyk, Milan R Vosko, Jorge Pagola, Eva Bartels, Stephan Schreiber, Felix Schlachetzki, Zsolt Garami, Andrei V Alexandrov, Natan M Bornstein, Elsa Azevedo, Radim Licenik, Janja Pretnar Oblak, Laszlo Olah, Joao Sargento Freitas, Uwe Walter, Branko Malojcic, Georgios Tsivgoulis

**Affiliations:** Stroke Center and Neurosonology Laboratory, Department of Neuroscience, University of Padua School of Medicine, Via Giustiniani 2, Padua, Italy; Stroke Center and Neurosonology Laboratory, Department of Neuroscience, University of Padua School of Medicine, Via Giustiniani 2, Padua, Italy; Stroke Center and Neurosonology Laboratory, Department of Neuroscience, University of Padua School of Medicine, Via Giustiniani 2, Padua, Italy; Department of Medical, Surgical, and Health Sciences, University of Trieste, Trieste, Italy; Department of Clinical Neurosciences, Neurology Clinic, Azienda Sanitaria Universitaria Giuliano Isontina, Trieste, Italy; Faculty of Medicine, Saarland University Hospital and Saarland University, Homburg, Saarland, Germany; Department of Neurology, Kepler University Hospital GmbH, Johannes Kepler University, Linz, Austria; Department of Neurology, Landesklinikum Amstetten, Amstetten, Austria; Vall d'Hebron University Hospital, Stroke Unit, Neurology Department, Barcelona, Spain; Neurology, Center for Neurological Vascular Diagnostics, Munich, Germany; Department Internal Medicine I, Kiel University, University Hospital Schleswig Holstein, Kiel, Germany; Department of Neurology, Center for Vascular Neurology and Intensive Care, medbo Bezirksklinikum Regensburg, University of Regensburg, Regensburg, Germany; Department of Cardiovascular Surgery, Houston Methodist Hospital, Houston Methodist DeBakey Heart & Vascular Center, Houston Methodist, Houston, Texas, United States of America; Department of Neurology, University of Arizona, Banner University Medical Center, Phoenix, AZ, United States; Department of Neurology, Shaare Zedek Medical Center and Faculty of Medicine, Hebrew University of Jerusalem, Shmuel Bait 12, Jerusalem 9103102, Israel; Department of Neurology, Centro Hospitalar Universitário de São João, Porto, Portugal; North West Anglia NHS Foundation Trust, Neurology and Cardiology Department, Edith Cavell Campus, Peterborough, Cambridgeshire, United Kingdom; Department of Vascular Neurology and Intensive Neurological Therapy, University Medical Centre Ljubljana, Ljubljana, Slovenia; Department of Neurology, Faculty of Medicine, University of Debrecen, Debrecen, Hungary; Department of Neurology, Unidade Local de Saúde de Coimbra, Coimbra, Portugal; Department of Neurology, Rostock University Medical Center, Rostock, Germany; Department of Neurology Zagreb School of Medicine, University Hospital Center Zagreb, Zagreb, Croatia; Second Department of Neurology, National and Kapodistrian University of Athens, School of Medicine, "Attikon" University Hospital, Athens, Greece

**Keywords:** stroke, ultrasound, point-of-care ultrasound

## Abstract

**Introduction:**

Early identification of stroke aetiology, hemodynamic monitoring and detection of complications represent key challenges for vascular neurologists. Stroke-point-of-care ultrasound (Stroke-POCUS) has emerged as a structured framework for integrating multimodal bedside ultrasound into stroke management.

**Patients and methods:**

Stroke-POCUS involves the comprehensive bedside use of various ultrasound modalities, including cervical and transcranial ultrasound, orbital ultrasound, echocardiography, venous system ultrasound, lung ultrasound, abdominal ultrasound and interventional ultrasound. These modalities are applied in an integrated manner to assess stroke patients in the acute setting, aiming to support diagnosis, etiological investigation, detection of complications and monitoring of treatment response, as an adjunct, not a substitution for computed tomography, magnetic resonance imaging, or standard comprehensive ultrasound examination.

**Results:**

The integration of multiple ultrasound modalities within Stroke-POCUS enables clinicians to obtain rapid, noninvasive answers to well-defined clinical questions at the patient’s bedside and in real time. This capability is particularly critical for patients requiring expedited diagnostics prior to urgent treatment initiation, for clinically unstable patients in whom intrahospital transport carries an increased risk of complications, as well as for assessing potential underlying causes, identifying secondary complications and monitoring treatment efficacy.

**Discussion and conclusion:**

Stroke-POCUS represents a comprehensive bedside imaging strategy that enhances the evaluation and management of stroke patients. By integrating multiple ultrasound techniques, it provides a more holistic view of stroke pathophysiology, complications and treatment monitoring, potentially improving clinical decision-making and individualised patient care.

## Graphical Abstract

**Graphical Abstract:**
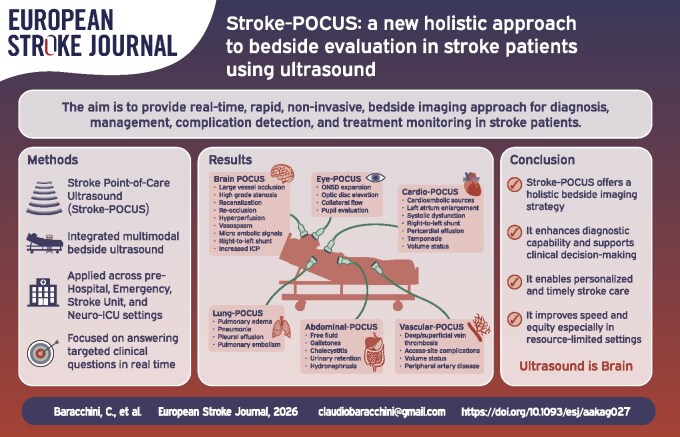

## Introduction

For decades, the mantra “Time is Brain”^1^ has guided acute stroke care. Every minute of delay in reperfusion therapy translates into the loss of millions of neurons, underscoring the urgency of rapid diagnosis and intervention. This principle has shaped emergency response systems, hospital workflows and even public awareness campaigns.

Yet, as technology advances, a new concept is emerging—one that emphasises not just speed, but precision: “Ultrasound is Brain.” In this framework, Stroke-POCUS does not replace traditional stationary neuroimaging such as CT or MRI. Instead, it complements these modalities, including advanced vessel and perfusion imaging, by providing rapid, bedside insights that enhance and extend diagnostic capability.

The emergence of Stroke-POCUS in general is rapidly redefining how clinicians assess and manage neurological emergencies. Thanks to major technological advances that have improved their precision and miniaturisation, portable ultrasound devices have moved beyond traditional radiology suites and now play a crucial role in the comprehensive management of acute stroke across its many stages (Tables S1–S3).

In 2022, a formal joint intersociety working group published a report introducing the concept of Neuro-POCUS.^2^ More recently, the World Organization of Neurosonology (WON) has developed multiple working groups on POCUS to validate this technique in stroke patients according to different settings:

## Pre-hospital assessment (ph-POCUS)

The implications of ph-POCUS are profound. While rapid transport to stroke centres remains essential, ultrasound introduces a layer of diagnostic precision previously unavailable in prehospital and emergency settings,^3^ allowing real-time visualisation of cerebral vasculature, brain parenchyma and hemodynamics at the bedside. It transforms “time” from a blunt metric into a nuanced, actionable understanding of brain physiology and perfusion**.** In other words, not all minutes are equal: knowing which regions of the brain are at risk, which vessels are occluded, and which tissue is salvageable can be more informative than a simple stopwatch. In addition, transcranial colour-coded duplex may allow the diagnosis of supratentorial intracerebral haemorrhage in prehospital settings.

Moreover, ultrasound democratizes access. In low-resource settings, a handheld ultrasound device can provide crucial information that might otherwise require advanced imaging. Before or during transport, ultrasound empowers clinicians to make timely, informed decisions, directing patients towards the most efficient pathway, thus bridging the gap between early recognition and a definitive therapy.^4^ Of course, ultrasound is not a replacement for speed—it is a complement. “Time is Brain” still holds true; minutes lost are neurons lost. But as stroke care evolves, we must recognise that ultrasound is brain as well, a tool that enables precision, guides therapy and ultimately saves neuronal function.

## In the emergency department (ed-POCUS)

Upon arrival to the Emergency, the patient undergoes a rapid stroke assessment, including a clinical examination (ABCs and NIHSS) and stationary neuroimaging (non-contrast CT, angio-CT, perfusion-CT) to guide treatment decision (intravenous thrombolysis or mechanical thrombectomy depending on imaging and time window). When immediate neuroimaging is unavailable, if the patient needs sedation for CT or MRI, or in patients with previous history of severe allergic reaction to contrast agents,^5^ cervical and transcranial vessel ultrasound can quickly detect large vessel stenosis or occlusion and signs of intracranial hypertension.^6-8^ In this context, ed-POCUS serves as an important indicator of timely and qualified care.^9,10^

## In the stroke unit/neurointensive care unit 

### Brain-POCUS (b-POCUS)

The first ultrasound evaluation in the Stroke Unit is represented by b-POCUS (Duplex sonography of the extra- and intracranial arteries), one of the most critical tools in Stroke-POCUS. It allows bedside, real-time evaluation of cerebral hemodynamics and vascular abnormalities: (1) to monitor arterial recanalization during systemic thrombolysis and/or endovascular treatment and provide prognostic information following the termination of acute reperfusion procedures^11,12^; (2) to rapidly exclude an atherosclerotic or non-atherosclerotic aetiology of stroke such as arterial dissection, temporal arteritis, Takayasu arteritis or fibromuscular dysplasia^13^; (3) to rapidly and reliably diagnose the presence and the degree of right-to-left shunt (eg. patent foramen ovale—PFO) as the cause of a paroxysmal cerebral embolism; (4) to detect the cause of an early neurological deterioration, such as arterial re-occlusion,^14^ microembolic signals,^15,16^ and hyperperfusion^17-19^; (5) to detect cerebral vasospasm and predict delayed cerebral ischemia in acute subarachnoid haemorrhage^20^; (6) to monitor cerebral autoregulation for blood pressure managment^21^; (7) to assess vasomotor reactivity and detect reversed Robin Hood phenomenon^22^; (8) to detect signs of supratentorial haemorrhage,^23^ or subdural hematoma^24^; (9) to detect signs of raised intracranial pressure: increased pulsatility indices,^25^ midline shift,^26-29^ third ventricle enlargement in hydrocephalus,^30,31^ undulations of the septum pellucidum^32^; (10) to assess the risk of post-stroke depression.^33^

### Eye-POCUS (i-POCUS)

The intracranial assessment is complemented by orbital ultrasound, a non-invasive technique allowing for the visualisation of the optic nerve and optic disc, where changes such increased optic nerve sheath diameter and optic disc elevation may suggest elevated intracranial pressure.^32,34,35^ In patients with a high-grade internal carotid artery stenosis or occlusion, orbital ultrasound also allows for assessment of the ophthalmic artery as an important secondary collateral to the brain. Additionally, the central retinal artery and vein can show evidence of arterial occlusion or venous thrombosis, which may be linked to ischemic stroke events.^36^ Orbital ultrasound comprises also an evaluation of the pupils. Pupillary ultrasound has been shown to be a safe and reliable technique for bedside pupillary function assessment, and a good alternative to infrared video pupillometry in patients with ocular trauma and severe eyelid swelling.^37^ In patients with carotid dissection, pupil reaction to light (photomotor reflex) is preserved, while pupil reaction to pinching at the base of the neck (cilio-spinal reflex) is compromised.^38^

### Cardio-POCUS (c-POCUS)

Once the evaluation of the cervical and intracranial vessels is completed, the focus shifts immediately to the heart, since a significant proportion of ischemic strokes result from cardioembolic events. Cardioembolic strokes are generally severe, and recurrence and mortality rates are high. Focused cardiac ultrasound (FoCUS), another critical component of Stroke POCUS validated for neurologists, plays a central role in identifying potential sources of embolism. FoCUS can screen heart pathologies to be detected by transesophageal echocardiography (TEE) or transthoracic echocardiography (TTE), such as left atrial enlargement and/or thrombus, left ventricular thrombus, left ventricular dilatation, PFO, valvular heart disease, endocarditis, cardiac tumours, complex aortic plaques and aortic dissection, all of which are known risk factors for stroke.^39^ The ability to assess the heart at the bedside through echocardiography is crucial in stroke patients, as identifying these underlying cardiac conditions can help guide treatment strategies such as anticoagulation therapy or surgical interventions.^40,41^ In addition, c-POCUS can be used to estimate right ventricular function and vena cava size to avoid hypovolaemia and volume overload, assess left ventricular ejection fraction, Takotsubo cardiomyopathy, hypertrophic cardiomyopathy, pericardial effusion and other signs of heart failure, which can increase stroke risk and predict worse acute stroke outcomes. This holistic evaluation helps neurologists understand the cardiac contributions to stroke aetiology and tailor management plans accordingly.

### Lung-POCUS (l-POCUS)

A comprehensive stroke assessment extends beyond the brain and heart to evaluate the respiratory system. Acute respiratory insufficiency represents a frequent and life-threatening complication following stroke. The most frequent pulmonary conditions on CT, in order of prevalence, include bronchitis/bronchiolitis (66.1%), atelectasis (66.1%), pleural effusion (60.6%), pneumonia (53%), pulmonary oedema (37.3%) and pulmonary artery embolism (27.5%). Bronchitis and bronchiolitis are independent risk factors for mortality. Early chest-ultrasound may help to identify patients at high risk for in-hospital mortality and guide appropriate treatment.^42^ L-POCUS can be used to assess for pulmonary oedema, pleural effusions, local or diffuse interstitial syndrome, or signs of right heart strain, conditions that are commonly seen in stroke patients due to immobility, cardiac dysfunction, or as complications of the stroke itself. The B-lines seen on chest ultrasound may indicate pulmonary congestion, which is critical in the management of stroke patients, particularly in those with compromised respiratory function or risk for aspiration. In addition, chest ultrasound can follow possible complications of central vein catheter insertion, such as pneumothorax and help in monitoring for potential stroke-related complications, such as pulmonary embolism (PE), which can occur as a sequela of immobilisation or coagulopathy, increasing the risk of poor outcomes.^43,44^ Having a bedside tool to detect these issues without needing to transfer the patient for imaging can be a game-changer in acute stroke care.

### Abdominal-POCUS (a-POCUS)

Stroke patients are often at high risk for abdominal complications, including gastrointestinal bleeding due to stress-induced ulceration (hemorrhagic gastritis), hepatic dysfunction due to congestive heart failure,^45^ splenic infarctions in cardioembolic stroke patients.^46^ Furthermore, stroke can disrupt autonomic control,^47^ leading to delayed gastric emptying, constipation, a decrease in gallbladder motility, potentially causing bile stasis, sludge, or gallstones. A common finding in stroke patients is a neurogenic bladder, where dysfunction of the nerves controlling the urinary bladder leads to urinary retention with possible hydronephrosis, or incontinence, or both. A-POCUS provides a non-invasive method for evaluating all these conditions,^44,48,49^ and identifying underlying disorders that may exacerbate stroke outcomes, such as liver cirrhosis or portal vein thrombosis. In some cases, a-POCUS may be used to: (1) monitor for aortic aneurysms or atherosclerotic plaques, that may predispose patients to embolic events; (2) investigate secondary hypertension: renal artery stenosis; polycystic kidney disease; adrenal masses; (3) manage anticoagulation complications: retroperitoneal, splenic or hepatic haemorrhages; (4) evaluate comorbidities (non-alcoholic fatty liver disease in metabolic syndrome; renal abnormalities with chronic kidney dysfunction) which increase stroke risk. By identifying these issues early, clinicians can make more informed decisions about the patient's care, including adjusting anticoagulation therapy and taking measures to address potential complications.

### Vascular-POCUS (v-POCUS)

Peripheral artery and vein ultrasound^50,51^ plays an important role in evaluating the vascular status of stroke patients. Peripheral artery disease (PAD) is a known risk factor for stroke, and evaluating the lower extremities for signs of atherosclerosis, thrombosis, or embolic disease can provide crucial insights into the overall vascular health of the patient. Additionally, deep vein thrombosis (DVT) and sometimes superficial vein thrombosis (SVT) are possible causes of stroke in patients with a right-to-left intracardiac shunt such as PFO. DVT is also a common complication in immobilised stroke patients and can lead to PE, further complicating the clinical picture.^52^ By using ultrasound to monitor for DVTs/SVTs and assess the peripheral circulation, clinicians can prevent secondary complications that could worsen patient outcomes.^53,54^ V-POCUS is also useful for detecting local complications of endovascular interventions, such as inguinal or femoral hematomas, pseudoaneurysms and arteriovenous fistulas.

### Interventional-POCUS (in-POCUS)

Using bedside ultrasound in stroke patients provides a highly effective, minimally invasive approach: (1) to insert peripheral/central venous catheters; (2) to control the correct position of naso-gastric tube and urinary catheter; (3) for procedures like lumbar puncture (spinal tap) or epidural injections; (4) to remove fluid from the pleural space (thoracentesis), the pericardial space (pericardiocentesis) or from the abdomen (paracentesis). By implementing these procedures, clinicians and staff nurses are better equipped to ensure a more efficient and safer patient care.^55,56^

## Discussion

Over the past decade, point-of-care ultrasound (POCUS) has emerged as a powerful tool for improving diagnostic timeliness and quality of care across diverse clinical settings.^56-61^ Its progressive integration into residency training programmes^62-65^ and proposals to consider POCUS the “fifth pillar” of the bedside physical examination^66^ underscore its growing clinical relevance.

**Figure 1:**
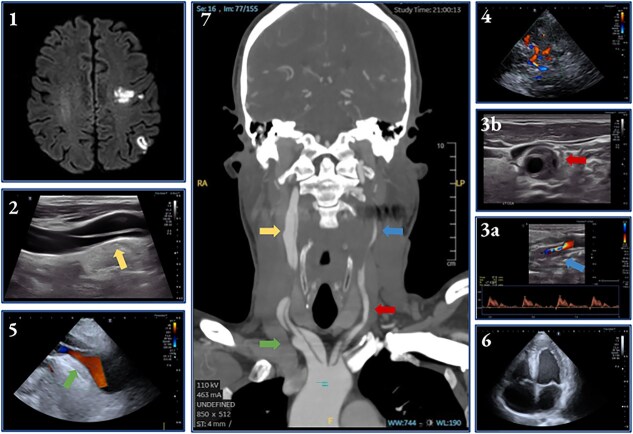
A 54-year-old man with a history of hereditary spherocytosis presented with mild aphasia and headache that began 24 hours earlier. Brain MRI showed subacute ischemic lesions in the left parietal region on diffusion-weighted imaging (1). Brain POCUS, performed in the stroke unit, revealed dissections in both common and internal carotid arteries, including a mobile dissecting membrane and a double lumen on the right side (2), along with a vessel wall hematoma (3a), and distal occlusion (3b). Transcranial Doppler showed reversed blood flow in the left A1 segment of the anterior cerebral artery indicating collateral circulation through the anterior communicating artery (4). Cardiac POCUS identified extension of the dissection from the brachiocephalic trunk (5) and features consistent with restrictive cardiomiopathy (6). CT angiography confirmed supraortic and cervical vessel findings (7).

**Figure 2:**
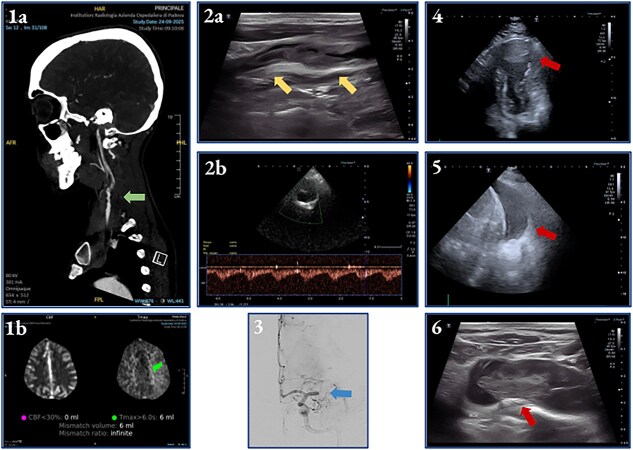
A 55-year-old woman with arterial hypertension and recent myocardial infarction woke up with acute right-sided weakness and facial drooping (NIHSS 4). Initial CT at 1 hour from stroke onset was normal, while CTA indicated distal common/internal carotid artery stenosis and a small hypoperfused area (1a–b). During IV thrombolysis, her neurological condition worsened (NIHSS 10). Brain-POCUS revealed a mobile thrombus in the left common/internal carotid artery with intracranial microembolic signals (2a–b) later confirmed by angiography showing carotid sub-occlusion and M1 segment occlusion (3). Endovascular thrombectomy achieved near-complete reperfusion (TICI 2c) , followed by carotid thromboaspiration and stenting. Cardio-POCUS, performed to investigate a possible cardiac embolic source, identified a left ventricular apical aneurysm with thrombus (4). Lung-POCUS performed for acute respiratory distress showed pleural effusion (5), while venous-POCUS detected subclavian vein thrombosis (6). High-resolution CT revealed lung cancer with pulmonary embolism, suggesting paraneoplastic hypercoagulability.

In this context, we introduce Stroke-POCUS, a novel paradigm defined by the integrated, bedside use of multimodal ultrasound across the prehospital, emergency department, stroke unit and neuro–ICU settings. Stroke-POCUS is explicitly designed to address time-sensitive, clinically actionable questions in real time throughout the stroke care continuum (Figures 1, 2). Although still evolving, this approach proved particularly valuable during the COVID-19 pandemic, enabling rapid bedside assessment, reducing reliance on advanced neuroimaging, shortening time to etiologic diagnosis and expanding access to diagnostic evaluation among stroke patients with SARS-CoV-2 infection.^5^

Ultrasound is well established as a cost-effective, safe and widely accessible imaging modality across neurological, cardiovascular, pulmonary, abdominal and vascular applications.^67-71^ Stroke-POCUS builds on these strengths by leveraging existing equipment and clinician expertise to deliver rapid bedside information during acute stroke evaluation. Compared with advanced imaging modalities such as CT angiography or TEE, Stroke-POCUS is relatively low cost, noninvasive, repeatable, and free of radiation or contrast exposure. Its targeted use may reduce diagnostic delays, expedite triage and treatment decisions—such as identification of large-vessel occlusion, cardiac embolic sources, or hemodynamic instability—and potentially limit unnecessary downstream testing. From a systems perspective, earlier and more focused decision-making may translate into shorter emergency department stays, improved imaging resource utilisation, and lower overall costs. These advantages are particularly relevant in prehospital and resource-limited settings, where access to advanced imaging is constrained.

Although Stroke-POCUS holds significant promise as a transformative approach, its implementation may initially raise concerns among stroke neurologists, largely reflecting the perceived burden of added responsibilities. Such concerns are understandable and mirror earlier experiences in emergency and critical care medicine, where ultrasound has since become embedded in routine practice. Stroke-POCUS is not intended to add tasks, but rather to facilitate earlier, more confident decision-making at the bedside. Importantly, the framework does not presume that individual stroke neurologists must independently master the full spectrum of ultrasound modalities at an expert level. Instead, Stroke-POCUS represents a conceptual, modular, and collaborative model, integrating ultrasound applications relevant to stroke care while respecting professional boundaries. It explicitly emphasises collaboration with cardiology, radiology, angiology, emergency medicine and EMS, ensuring that complex or uncertain findings are escalated appropriately. Stroke-POCUS is therefore designed to complement—not replace—established ultrasound specialties.

To operationalise this approach, we propose a “core minimum” Stroke-POCUS set (Table 1), aimed at standardising practice when ultrasound is performed primarily by stroke physicians. This focused set comprises high-yield examinations that are feasible with appropriate training, directly inform acute decision-making and include clear referral pathways for abnormal or inconclusive findings. Defining this scope clarifies responsibilities, facilitates interdisciplinary agreement on indications and reporting, supports structured training and credentialing, and enhances patient safety by distinguishing screening assessments from comprehensive diagnostic studies. More advanced examinations remain within the remit of collaborating specialties, ensuring complementary rather than overlapping roles.

**Table 1:** Core minimum stroke-POCUS set.

**US modality**	**Specific clinical question** **“What” to examine**	**Stroke-POCUS operator** **“Who” examines**
**ph-POCUS**	TCCD: Large Vessel Occlusion?	EMS physician
**ed-POCUS**	Cervical duplex US: Carotid/Vertebral occlusion? Carotid/Vertebral high-grade stenosis? TCCD: Large Vessel Occlusion? Large Vessel high-grade stenosis?	Stroke physician ED physician
**Stroke-Unit POCUS**		
**b-POCUS**	Cervical duplex US: High-grade stenosis or occlusion? Vulnerable plaque? Dissection? Vasculitis? TCCD/TCS: Complete/partial recanalization? Re-occlusion? Hyperperfusion? Activation of collaterals? MES (ipsilateral, bilateral)? Right-to-left shunt? Cerebral haemorrhage? Vasospasm? Midline shift/diffuse increase of Pulsatility index (↑ ICP)? Increased ventricle width (hydrocephalus)?	Stroke physician
**i-POCUS**	Activation of collaterals (inverted flow in OA)? ONSD expansion (↑ ICP)? Optic disc elevation (↑ ICP)? Absent Cilio-spinal Reflex (Carotid dissection)?	Stroke physician
**c-POCUS**	Identify cardioembolic sources: Intracardiac thrombus? Left atrium enlargement (AF surrogate)? Intracardiac tumour (eg. myxoma)? Identify hemodynamic instability: Severe LV dysfunction or acute heart failure? New regional wall-motion abnormality (suggesting acute MI)? Large pericardial effusion/tamponade?	Stroke physician
**l-POCUS**	Identify causes of respiratory distress: Pulmonary oedema (cardiogenic)? Pneumonia? Pleural effusion? Pulmonary embolism?	Stroke physician
**a-POCUS**	Urinary retention?	Stroke physician
**v-POCUS**	Deep/superficial vein thrombosis? IVC diameter (hypovolaemia? congestion?)?	Stroke physician
**IN-POCUS**	Imaging during insertion of peripheral venous catheter Imaging during insertion of central venous catheter Check position of nasogastric tube Check position of urinary catheter	Stroke Nurse Stroke physician

Abbreviations: AF, atrial fibrillation; B-POCUS, brain point-of-care ultrasound; C-POCUS, cardiac point-of-care ultrasound; ED, emergency department; ED-POCUS, emergency department point-of-care ultrasound; EMS, emergency medical services; ICP, intracranial pressure; I-POCUS, intracranial point-of-care ultrasound; IN-POCUS, interventional point-of-care ultrasound; IVC, inferior vena cava; L-POCUS, lung point-of-care ultrasound; LV, left ventricular; MES, microembolic signals; MI, myocardial infarction; OA, ophthalmic artery; ONSD, optic nerve sheath diameter; PH-POCUS, pre-hospital point-of-care ultrasound; POCUS, point-of-care ultrasound; TCCD, transcranial colour-coded Doppler; TCS, transcranial sonography; US, ultrasound; V-POCUS, vascular point-of-care ultrasound.

The WON has established dedicated working groups to validate Stroke-POCUS applications across diverse clinical environments using a structured, competency-based training framework. Training progresses from foundational knowledge (ultrasound physics, knobology, artefacts, and safety principles) to supervised hands-on scanning across cerebral, cardiac, pulmonary, abdominal, and vascular applications, and culminates in clinical integration focused on real-time decision-making. Competency is assessed through documented examinations, direct observation, image review, and practical and written evaluations. Given the operator-dependent nature of POCUS, ongoing quality assurance, continuing education, and image review are essential.^72-74^ Emerging artificial intelligence (AI) tools are expected to further enhance training, standardisation, and dissemination,^75,76^ particularly in prehospital, mobile stroke unit, and resource-limited settings. Stroke-POCUS is deliberately structured around minimal scan sets, tiered competencies, and parallel workflows that integrate seamlessly into existing stroke pathways without delaying definitive imaging or treatment, prioritising speed, reproducibility, and safety over comprehensive imaging.

Beyond training, the cost-effectiveness of Stroke-POCUS depends critically on thoughtful integration into existing workflows. While early data are encouraging, robust, context-specific economic analyses remain necessary. The scope of Stroke-POCUS should therefore be tailored to local resources and clinical priorities. In resource-limited or community hospitals, transcranial Doppler–based screening for large-vessel occlusion may offer the greatest value for early triage and transfer decisions. In primary stroke centres, carotid duplex screening and recanalisation monitoring may optimise thrombolysis and interfacility transfer. In comprehensive stroke centres, advanced applications—including vasospasm surveillance, emboli detection, post-reperfusion hemodynamic assessment, and noninvasive intracranial pressure monitoring—may further individualise care. Across all settings, Stroke-POCUS is most effective when purpose-driven, context-specific, protocolised, and competency-based, functioning as a rapid physiologic extension of the bedside examination rather than a substitute for definitive imaging.

Finally, although POCUS is noninvasive, it is not risk free. Diagnostic accuracy is highly operator dependent, and inappropriate use may lead to diagnostic error or delays in care.^77,78^ Users must understand technical limitations—such as inadequate acoustic windows—and recognise when comprehensive imaging is required. As a bundled strategy, Stroke-POCUS shares these limitations, along with challenges related to workflow integration and uncertainty regarding its incremental benefit over established imaging pathways. Addressing these issues will require targeted education, supervised training, robust quality assurance, access to appropriate devices, and protected time for clinical implementation.^44,79-81^ AI-assisted ultrasound acquisition and interpretation—now increasingly integrated into commercial ultrasound platforms^82^—has the potential to substantially lower technical barriers to Stroke-POCUS implementation. Emerging evidence demonstrates robust diagnostic performance across multiple neurovascular and cardiocerebral applications. Ongoing prospective studies are evaluating AI-assisted transcranial duplex sonography for early intracerebral haemorrhage detection,^83^ while AI-based algorithms have achieved diagnostic accuracies of approximately 90% for internal carotid artery stenosis in acute stroke^84^ and 85%–92% for middle cerebral artery vasospasm across diverse etiologies.^85^ Additional developments include deep learning–based optic nerve sheath diameter measurement for noninvasive detection of elevated intracranial pressure.^86-88^ Beyond neurosonology, AI guidance has consistently enhanced point-of-care echocardiography, improving recognition of systolic and diastolic dysfunction by ultrasound-trained emergency physicians^89^ and enabling diagnostically interpretable cardiac imaging by inexperienced users in the majority of cases.^77,90-92^ These data underscore the transformative potential of AI-assisted ultrasound to expand the reliability, scalability, and clinical impact of Stroke-POCUS when performed by operators with basic ultrasound training. Collectively, these measures are essential to ensure safe, effective, and sustainable adoption of Stroke-POCUS in contemporary stroke care.

## Conclusion

The future of stroke care lies at the intersection of urgency and insight. Incorporating Stroke-POCUS into the management of stroke patients represents a significant advancement in acute stroke care. By utilising a combination of different ultrasound techniques, clinicians gain a comprehensive, real-time view of the patient’s condition. With dedicated training, this multimodal approach allows for better identification of stroke causes, early detection of complications, and more targeted interventions—all at the bedside. As the evidence continues to grow, even with the aid of artificial intelligence, Stroke-POCUS is poised to become an integral part of acute stroke management, improving outcomes and reducing the burden of this devastating disease.

## Supplementary Material

aakag027_Supplemental_Files
