# Supplementary material for: Stroke-point-of-care ultrasound: a new holistic approach to bedside evaluation in stroke patients using ultrasound
Source: Eur Stroke J. 2026 Apr 7;11(4):aakag027. doi: 10.1093/esj/aakag027 (PMC13131222; doi:10.1093/esj/aakag027)
Supplement: aakag027_Supplemental_Files [file aakag027_supplemental_files.zip › 3_eTables_-_STROKE-POCUS__copia.docx]

**eTable 1: SEARCH FOR EMBOLIC/THROMBOTIC STROKE CAUSES**

| **POCUS** | **Clinical question** | **Ultrasound findings** |
| --- | --- | --- |
| **Carotid/Vertebral POCUS** | Is there a cervical vessel occlusion responsible for the stroke symptoms?  Is there a cervical vessel stenosis responsible for the stroke symptoms?  Is there a rare vessel abnormality responsible for the stroke symptoms?  Is there a dynamic cervical vessel stenosis/occlusion responsible for the stroke symptoms?  Are there supportive signs of an inflammatory etiology for the stroke symptoms? | Acute carotid/vertebral artery occlusion: i) atherothrombotic; ii) dissective; iii) embolic; iv) inflammatory or vasculitic; v) radiation-induced.  Acute carotid/vertebral artery stenosis: i) atherothrombotic; ii) dissective; iii) embolic; iv) inflammatory or vasculitic; v) radiation-induced.  Grading of stenosis (moderate:>50%; severe: >70%).  High-Risk Plaque (echolucency, surface irregularity, ulcer, large necrotic core, intraplaque hemorrhage, intraplaque neovascularization, floating thrombus).  Carotid web; fibromuscular dysplasia; vascular tumors.  Eagle’s disease; Bow-Hunter disease.  Superficial Temporal Artery/Occipital Artery stenosis/occlusion. Halo sign. Non-compressible temporal artery. |
| **Transcranial POCUS** | Is there an intracranial vessel occlusion/stenosis responsible for the stroke symptoms?  What is the impact of cervical vessel occlusion/stenosis on cerebral hemodynamics?  Are there signs of diffuse disease?  Are there signs supportive of a paradoxical embolism?  Are there signs of a reversible etiology for the stroke? | Acute large vessel occlusion/stenosis.  Grading of stenosis (>50%).  Distal flow disruption: minimal, blunted, dampened.  Activation of Collaterals: AcomA, AcomP, leptomeningeals, OA.  Microemboli detection.  Vasomotor Reactivity impairment. Reversed Robin Hood phenomenon.  Multifocal and/or multivessel stenoses/occlusions.  Detection and quantification of a right-to-left shunt.  Reversible Cerebral Vasoconstriction Syndrome. Reversible infectious or inflammatory arteriopathies. Post-SAH or post-traumatic vasospasm. Drug- or toxin-induced vasospasm. Migraine-related vasospasm. Fibromuscular dysplasia – dynamic component. |
| **Eye-POCUS** | Are there supportive signs of atheroembolic/vasculitic mechanism for the stroke symptoms?  Are there supportive findings of carotid artery dissection?  Is there an impact of cervical vessel occlusion/stenosis? | Central retinal artery occlusion. Retrobulbar spot sign.  Posterior ciliary artery occlusion.  Abnormal pupillary reflex patterns.  Retrograde flow in OA. |
| **Cardio-POCUS** | Is there a cardiac source of embolism that indicates early anticoagulation or surgical/endovascular treatment?  Is the etiology of stroke consistent with possible septic cardiac embolism requiring antibiotic therapy and/or surgical intervention?  Are there signs supportive of a paradoxical embolism? | Atrial or ventricular thrombi.  Cardiac tumors.  Suspected cardioembolic mechanism in patients with left atrial enlargement.  Complex aortic plaques.  Aortic dissection.  Endocarditis (infected masses or vegetations on heart valves).  Valvular dysfunction.  Detection and quantification of a right-to-left shunt. Identification of intracardiac or extracardiac sources of paradoxical embolism.  Supplementary information includes interatrial septum characteristics. |
| **Vascular-POCUS** | Are there signs supportive of a paradoxical embolism? | Deep vein thrombosis.  Superficial vein thrombosis. |

**eTable 2: EARLY DETECTION OF NEUROLOGICAL COMPLICATIONS OF ACUTE BRAIN DAMAGE**

|  | **Clinical question** | **Ultrasound findings** |
| --- | --- | --- |
| **Brain-POCUS** | In patients who underwent intravenous thrombolysis and/or endovascular treatment, how is the previously occluded vessel?  After reperfusion therapies for ischemic stroke, is the patient at risk of hemorrhagic transformation or poor outcome?  Are there signs of intracranial hemorrhage?  Is the patient with SAH or RVCS at risk of delayed cerebral ischemia?  Are there signs of increased intracranial pressure (ICP)? | Recanalization of occluded artery.  Partial recanalization of occluded artery.  Re-occlusion.  Hyperperfusion.  Microembolization in cerebral arteries.  Malignant brain edema.  Intracerebral hemorrhage (ICH) or subdural hematoma (SDH) responsible for the stroke symptoms.  Hemorrhagic transformation (HT) of the ischemic region.  Intracranial bleeding remote from the infarcted area.  Monitoring hematoma size: expansion/resorption.  Detection and grading of vasospasm.  Increased resistance to flow (diffuse increase of Pulsatility Index values).  Midline shift.  Third ventricle enlargement.  Undulations of the Septum Pellucidum. |
| **Eye-POCUS** | Are there signs of ICP?  Are there signs supportive of optic nerve or retinal damage? | Increased optic nerve sheath diameter (ONSD expansion).  Optic disc elevation (ODE): papilloedema.  Marcus Gunn Pupil (Relative Afferent Pupillary Defect - RAPD). |

**eTable 3: SYSTEMIC COMPLICATIONS AND PROCEDURES**

| **POCUS** | **Clinical question** | **Ultrasound findings** |
| --- | --- | --- |
| **Cardio-POCUS** | Is there a cardiac cause of respiratory distress syndrome?  Are there signs of myocardial damage?  Does POCUS help clinicians optimize blood pressure, heart rate, and fluid management in patients with acute ischemic stroke?  Is there a pericardial effusion? | Diffuse B-lines on lung ultrasound, pleural and/or pericardial effusion, and inferior vena cava diameter increase with reduced collapsibility suggest heart failure-related pulmonary congestion.  Right ventricular dysfunction, right atrial and/or ventricular thrombus, in combination with A-lines/normal lung echography or signs of cortical infarction, and deep and/or superficial vein thrombosis might indicate pulmonary embolism.  Left ventricle dysfunction: reduced ejection fraction.  Regional/global wall motion abnormalities due to myocardial infarction or neurogenic stress cardiomyopathy, Takotsubo cardiomyopathy.  Readily detect significant hemodynamic valvular stenosis or regurgitation, increased left ventricular wall thickness affecting ejection fraction, and altered left and right ventricular volumes/functions, all of which may influence stroke prognosis.  Right ventricular function and Inferior vena cava diameter and collapsibility provide key information on volume status, to avoid hypovolemia and volume overload.  Pericardial effusion caused by local or systemic causes (autoimmune, neoplastic, or oncotic) can impair diastolic function. |
| **Vascular-POCUS** | What is the cause of acute limb pain and edema?  Is there a local complication of endovascular intervention?  Are there signs supportive of peripheral vein disease?  Are there signs supportive of peripheral artery disease (PAD)? | Deep vein thrombosis.  Superficial vein thrombosis.  Hematoma.  Stenosis/occlusion of the femoral/radial artery.  Pseudoaneurysm. Arteriovenous fistula.  Chronic Venous Insufficiency.  Venous stasis.  Atherosclerotic plaques causing stenosis or occlusion of peripheral arteries. |
| **Lung-POCUS** | What is the cause of respiratory distress syndrome? | A combination of echocardiographic evidence of altered right ventricular volume/function, the presence of right atrial and/or ventricular thrombus, A-lines/normal lung echography or signs of cortical infarction, and deep and/or superficial vein thrombosis might indicate pulmonary embolism.  Diffuse B-lines, pleural and/or pericardial effusion, and inferior vena cava diameter increase with reduced collapsibility suggest heart failure-related pulmonary congestion.  Focal B-lines and/or lung consolidation, with or without pleural effusion, suggest pneumonia or pulmonary atelectasis. |
| **Abdominal-POCUS** | What is the cause of acute abdominal pain?  Are there signs of autonomic dysfunction?  Are there signs of congestive heart failure?  Are there signs of significant abdominal comorbidity?  Are there signs of secondary hypertension? | Acute urinary retention, including possible urinary catheter displacement.  Post-void residual urine.  Bladder wall thickening/diverticula/stones  Hydroureter. Hydronephrosis.  Renal stones.  Gallbladder stones.  Gallbladder dilation with wall thickening (Cholecystitis).  Bleeding: gastric, hepatic, splenic, retroperitoneal hemorrhage.  Splenic infarction.  Aortic aneurysm rupture.  Aortic dissection.  Gastric stasis.  Bile stasis and gallbladder sludge.  Reduced Intestinal motility (Ileus or pseudobstruction).  Hepatomegaly with dilatation of suprahepatic veins.  Hepatic cirrhosis; steatosis.  Portal vein thrombosis.  Renal artery stenosis.  Polycystic kidney disease.  Adrenal mass. |
| **Interventional-POCUS** | Is venous access difficult?  Is the nasogastric tube/urinary catheter correctly positioned?  Is there a need for fluid removal?  Is there a need to perform a lumbar puncture or an epidural injection in a difficult situation? | Help insert peripheral/central venous catheters.  Control the correct position of naso-gastric tube and urinary catheter;  Help to remove fluid from the pleural space (thoracentesis), the pericardial space (pericardiocentesis) or from the abdomen (paracentesis).  Help perform a lumbar puncture or epidural injections in difficult patients. |
