# Supplementary material for: Stroke-point-of-care ultrasound: a new holistic approach to bedside evaluation in stroke patients using ultrasound
Source: Eur Stroke J. 2026 Apr 7;11(4):aakag027. doi: 10.1093/esj/aakag027 (PMC13131222; doi:10.1093/esj/aakag027)
Supplement: aakag027_Supplemental_Files [file aakag027_supplemental_files.zip › Risposte finali.docx]

Reviewer(s)' Comments to Author:

Reviewer: 1

Comments to the Author
I thank the authors for the clarifying answers and additional changes following my comments. I have no other comments.

We thank the reviewer for his insightful suggestions, which have enabled us to substantially expand and clarify the manuscript.

Reviewer: 2

Comments to the Author
Thank you for the thorough revision adding and explaining the major points.
We sincerely thank the reviewer for his careful reassessment and for the opportunity to further clarify the conceptual framework of Stroke-POCUS. Your comments have helped us to further refine and strengthen the manuscript. We address each point below.

Q) Please be more concise in the abstract introduction

Sentences like: Stroke remains one of the leading causes of death and long-term disability worldwide. Timely diagnosis, continuous monitoring, and effective management of acute stroke are critical to improve outcomes in stroke patients are too generalized for an abstract of a Stroke journal.

A) We agree that the opening of the abstract was overly broad for a specialized stroke readership. The abstract has been revised accordingly to better align with the target audience (see Abstract).

Q) In the introduction " Neuro-POCUS" is described as a new and eventually revolutionary concept. Even the emphasis is understandably given for the scope of the article, POCUS is not a new concept and is performed in EMDs all over the world. So this might be seen in the context of a general evolvement of ultrasound in the acute setting.

A) We fully agree that the POCUS concept is well established in emergency and internal medicine. Only recently it has been adopted in Neurology as Neuro-POCUS. The novelty of our manuscript lies in a further conceptual advance, specifically the application of POCUS to acute stroke management, which we propose as Stroke-POCUS. As further outlined in our reply to the next point of the reviewer, we have adjusted some content of the Table according to the reviewer’s suggestions. We are confident that we have now appropriately refined and more clearly explained the concept of Stroke-POCUS.

Q) Thank you for adding a core minimum of training for Stroke-POCUS but this is more reflecting the maximum. Since it would be more realistic to stepwise increase a core minumum, I would advice to choose first the most important parts (ultrasound is brain).

EMD doctors and experts in internal medicine are usually trained in other modalities so it is questionable a pulmonologist is needed to confirm a lung-pocus, likewise the abdominal POCUS be confirmed by a gastroenterologist.

Please consider if diagnostic skills on the level of including /excluding pulmonary embolism during lung- POCUS. or excluding a cholecystitis during the abdominal- POCUS is a realistic level of ultrasound skill for a stroke physician.

The search for stroke etiologies should be a major point. Please reflect if lung, abdominal POCUS should be part of the CORE minumum.

A) After carefully considering the reviewer’s comments, we have revised the Table outlining the Core minimum components of Stroke-POCUS. Specifically: 1. we removed the last column indicating potential confirmatory scan operators; 2. stroke etiologies represent a major pillar in the table; 3. neurological complications represent another major point of core minimum (ultrasound is brain); 4. we excluded some internistic complications (eg. abdomen) from the Core minimum. The supplementary tables, which present a more advanced and comprehensive framework, were not modified. This modular structure is designed to be scalable and adaptable across different clinical settings, facilitating implementation while preserving the conceptual foundation of Stroke-POCUS (see Table).
